# Apples and Oranges: Comparing High-definition Intravascular Ultrasound and Optical Coherence Tomography

**DOI:** 10.1016/j.jscai.2025.103574

**Published:** 2025-05-01

**Authors:** Zubeen Azari, John J. Lopez

**Affiliations:** Loyola University Medical Center and Loyola Stritch School of Medicine, Hinsdale, Illinois

**Keywords:** coronary intervention, intravascular imaging, intravascular ultrasound, optical coherence tomography

Advanced intravascular imaging (IVI) improves the success of complex percutaneous coronary intervention (PCI), as precise imaging has been demonstrated to assist in decision making and improve outcomes.^1^ The use of IVI is associated with lower in-hospital mortality, myocardial infarction, and target lesion revascularization than angiographically-guided PCI.^1^, ^2^, ^3^, ^4^ Utilization of IVI has become more common in the United States, with intravascular ultrasound (IVUS)-guided PCI increasing by >300% from 2008 to 2019 and optical coherence tomography (OCT)-guided PCI increasing by >500% from 2011 to 2019^5^; however, overall adoption of these technologies remains relatively low at approximately 15% of PCI procedures in the United States in 2019, with median operator use at only 3.92% despite current guidelines supporting a class IIA indication.^4^^,^^6^

IVUS and OCT provide superior visualization than coronary angiography, improving the procedural decision making that affects outcomes, yet the technologies have notable differences. Although both modalities allow high-resolution cross-sectional imaging resulting in the ability to assess lesion length, appropriate stent sizing, malapposition, suboptimal stent sizing, and residual dissection, IVUS can provide full vessel imaging, allowing more consistent assessment of vessel size, deep wall plaque features, and plaque burden, whereas OCT offers higher resolution imaging, resulting in the ability to identify residual dissection, malapposition, and plaque morphology with greater clarity. Further iterations and improvements in the technologies continue, including the development of high-definition (HD)-IVUS (>45 MHz), which provides improved resolution over traditional IVUS, making the choice of which IVI modality to select even more difficult for the practicing interventionalist.^7^

In this issue of *JSCAI*, Wu et al^8^ directly compare the ability of HD-IVUS and OCT to assess lumen size and plaque morphology. In this analysis, the authors compared in vitro HD-IVUS and OCT findings from 8 silicone models of coronary arteries with microcomputed tomography (μCT) imaging, denoted as the “ground truth.” Mean lumen diameter (MLD) and lumen area were measured and compared with μCT, demonstrating that HD-IVUS overestimated MLD (+0.06 ± 0.05 mm; error = +1.7%) and lumen area (+0.28 ± 0.34 mm^2^; error = +2.5%), and OCT underestimated MLD (0.17 ± 0.06 mm; error = −4.7%) and lumen area (−1.2 ± 0.72 mm^2^; error = −10%). Additionally, an in vivo analysis was performed with sequential HD-IVUS and OCT of 12 native coronary arteries, which demonstrated no significant difference between MLD and lumen area.

Wu et al^8^ are to be commended for this well-performed and timely comparison of the current versions of HD-IVUS and OCT imaging, which is important in helping to evaluate the incremental improvements being made with IVI technologies. Additionally, this work demonstrates the industry’s continued commitment to these modalities, despite the disconnect between the clinical utility and overall adoption of these technologies. To summarize, the authors reported on the difference in lumen diameter and lumen area measurements between the most advanced versions of OCT and IVUS and demonstrated that HD-IVUS overestimates dimensions to a smaller degree than OCT overestimates them. Although these differences are statistically significant and interventionalists using these modalities should be aware of the direction and magnitude in measurement differences between them, these submillimeter differences are not clinically relevant.

There are additional limitations to this work. First, the data obtained from silicone models used μCT as ground truth; however, there is no “ground truth” for the clinical data provided. Second, extrapolating findings from the silicone models to in vivo measurements fails to account for many factors, including blood clearance, lack of contrast use, vascular tone, and how the imaging tool and its resolution are able to assess the leading edge of biologic tissue at the lumen vessel interface.^9^ Third, the comparison of the ability of HD-IVUS and OCT to identify normal vessel structures and certain pathologic morphologies is solely descriptive and does not add to our understanding of the differences between these modalities. Most importantly, this analysis does not provide data or context to conclude that the differences in lumen dimensions and area measurements between HD-IVUS and OCT has clinical relevance or impact.

HD-IVUS is a welcome advance to the interventionalist’s imaging arsenal. Along with OCT, HD-IVUS can provide accurate assessment of coronary lumen dimensions and likely enhanced identification of plaque and vessel morphology. The practicing interventionalist should be familiar with the advantages and disadvantages, as well as the differences, between these modalities, recognizing that measurement differences between them, while real, are unlikely to affect clinical decision making and that both tools are far superior to angiography in guiding coronary interventions. It is this last point that requires further emphasis, as the more important issue in the field of IVI should not be a focus on minor differences in the measurement capabilities of these technologies but instead how to increase routine usage and adoption of these tools.
